# Regression models for analyzing radiological visual grading studies – an empirical comparison

**DOI:** 10.1186/s12880-015-0083-y

**Published:** 2015-10-30

**Authors:** S. Ehsan Saffari, Áskell Löve, Mats Fredrikson, Örjan Smedby

**Affiliations:** Department of Medical and Health Sciences (IMH), Linköping University, Linköping, Sweden; Sabzevar University of Medical Sciences, Sabzevar, Iran; Department of Diagnostic Radiology, Lund University, Clinical Sciences, Lund, Sweden; Department of Radiology, Landspitali University Hospital, Reykjavik and Faculty of Medicine, University of Iceland, Reykjavik, Iceland; Department of Clinical and Experimental Medicine, Linköping University, Linköping, Sweden; KTH Royal Institute of Technology, School of Technology and Health, Alfred Nobels allé 10, SE-141 52 Huddinge, Stockholm Sweden

**Keywords:** Image quality, Visual grading, Ordinal data, Regression models, Fixed effects, Random effects

## Abstract

**Background:**

For optimizing and evaluating image quality in medical imaging, one can use visual grading experiments, where observers rate some aspect of image quality on an ordinal scale. To analyze the grading data, several regression methods are available, and this study aimed at empirically comparing such techniques, in particular when including random effects in the models, which is appropriate for observers and patients.

**Methods:**

Data were taken from a previous study where 6 observers graded or ranked in 40 patients the image quality of four imaging protocols, differing in radiation dose and image reconstruction method. The models tested included linear regression, the proportional odds model for ordinal logistic regression, the partial proportional odds model, the stereotype logistic regression model and rank-order logistic regression (for ranking data). In the first two models, random effects as well as fixed effects could be included; in the remaining three, only fixed effects.

**Results:**

In general, the goodness of fit (AIC and McFadden’s Pseudo *R*^2^) showed small differences between the models with fixed effects only. For the mixed-effects models, higher AIC and lower Pseudo *R*^2^ was obtained, which may be related to the different number of parameters in these models. The estimated potential for dose reduction by new image reconstruction methods varied only slightly between models.

**Conclusions:**

The authors suggest that the most suitable approach may be to use ordinal logistic regression, which can handle ordinal data and random effects appropriately.

## Background

When evaluating medical imaging methods, the most relevant performance measures of a procedure are related to its ability to produce correct answers to a diagnostic problem. This is typically done with concepts such as sensitivity, specificity and receiver operating characteristic (ROC) analysis. When developing a new method, however, it is often necessary to fine-tune numerous parameters that need to be specified in modern imaging equipment in order to obtain as much diagnostic information as possible at the minimum cost in radiation dose (effective dose) to the patient. In this optimization process, a common approach is to perform *visual grading experiments*, where a group of observers (e.g. radiologists) assess the fulfillment of certain well-defined image quality criteria using an ordinal scale [1]. As the data are given on an ordinal scale, the data analysis methods should be chosen accordingly, using techniques that are appropriate for such data. Still, a number of studies have been published where ordinal data from visual grading experiments are analyzed with ANOVA and similar linear models, although these build on assumptions of interval scale data, homoscedasticity and so forth.

In earlier publications, our group has proposed to use ordinal regression models in these situations to compare alternative imaging procedures [2]. Using such models, and an assumption of the relationship between the effective dose to the patient and the image quality, it is also possible to estimate the potential for dose reduction that may be expected when a new technique is introduced [3]. Based on an experiment where both the imaging technique and the effective dose are varied, the estimated dose reduction is obtained from the ratio between two regression coefficients in the regression equation. Since two of the experimental factors, the patient and the observer, are not interesting *per se*, but can be seen as samples from two underlying populations, it may be appropriate to treat them as random effects, which can also be done with ordinal regression models [4].

In addition to the most common form of ordinal regression, the proportional odds model [5], alternative approaches for analyzing ordinal data with regression models include the partial proportional odds model [6] and the stereotype logistic model [7]. These do not seem to have been applied to visual grading data before. In addition, random effects models have not been systematically compared to models with only fixed effects. Finally, it is not known to what extent the results of ordinal regression models differ from those of the simpler linear models.

Thus, the aim of the present study was to review regression models potentially suitable for analyzing visual grading studies and to empirically compare them on already available data, in particular to study the effect of including random effects in the model.

## Material and methods

### Data

The data used were taken from a previously published study on image quality and radiation dose in brain Computed Tomography (CT) which evaluated two new reconstruction algorithms, i.e. methods for creating images from the acquired raw data [8]. It has been suggested that new reconstruction algorithms (in particular iterative algorithms) may improve image quality to such an extent that the radiation dose to the patient may be reduced without impairing the image quality, which otherwise occurs when the radiation dose is reduced. Six neuroradiologists evaluated image quality in images acquired from 40 patients, each of whom underwent two consecutive brain CT examinations with two different effective dose levels. Images from all 80 examinations were reconstructed using four different image reconstruction methods: the traditional filtered back projection algorithm using the full dose (CTDI_vol_) of 57 mGy (*fd*), which served as the reference, the same algorithm using a reduced dose of 40 mGy (*rd*), and two different levels of iterative reconstruction algorithms (*id2* and *id4*), also using the reduced dose. In the visual evaluation, each observer individually graded three image quality criteria – gray-white-matter discrimination (GW), basal ganglia delineation (BG) and general image quality (GQ) – using a four-grade ordinal scale ranging from 1 (poor) to 4 (excellent). In addition, each observer ranked each set of four reconstructions, i.e. sorted the four image stacks in order from 1 (best) to 4 (worst) for each of the image quality criteria.

Thus the grading data comprises 3 image quality scores (*GWscore*, *BGscore* and *GQscore*) and 3 image quality ranks (*GWrank*, *BGrank* and *GQrank*) for each imaging protocol, observer and patient. As there were 6 observers and 40 patients, and we considered 4 imaging protocols (*nd*, *rd*, *id2* and *id4*), the dataset consists of 6 × 40 × 4 = 960 observations. The data were stored in *Stata* format, and *Stata* 13.1 (StataCorp, College Station, TX, USA) was used for all analyses.

The ethical approval of the acquisition of data for the original publication [8] was given by the regional research ethics committee in Lund, Sweden (decision nr. 2010/594, date Nov. 11, 2010). Written informed consent was obtained from each patient before examination, and the study was performed in compliance with the Helsinki Declaration.

### Analysis of absolute grading scores

In this section, different regression models will be discussed. In all models, the response variable is *GWscore*, which is treated as an interval scale variable. We assume that the influence of dose is best modeled via the logarithm of the dose rather than the dose itself [2]. Thus, there are five covariates in the regression models: log(*CTDI*), *id*2*, id*4, patient and observer, the two last of which are nominal, whereas *id2* and *id4* are dummy variables indicating whether an iterative reconstruction method was used.

#### Regression models with fixed effects

We suppose in this section that all covariates are fixed effects in the regression models. We start the analysis with the most fundamental regression model, i.e. the linear regression model, and will then discuss the logistic regression models, which are the main concern of this paper.

##### Linear model

In a linear regression model, it is supposed that the relationship between the dependent variable and the vector of regressors is linear; thus the model takes the following form:

1 $$ GWscore={\beta}_0+{\beta}_1\  \log (CTDI)+{\beta}_2\  id2+{\beta}_3\  id4+{\beta}_{4,p} + {\beta}_{5,o}+\epsilon $$

where β_i_’s are the regression coefficients, and ϵ is an error term from the population. This was achieved with the following Stata command:

$$ regress\  GWscore\  logCTDI\  id 2\  id 4\ i. patient\ i. observer $$

##### Ordinal logistic regression

The ordinal logistic regression model (proportional odds model) is used when the dependent variable is ordinal. The cumulative probability of this regression model can be expressed in this form:

2 $$ P\left( GWscore\le i\;\Big|\boldsymbol{x}\right)=\frac{e^{\beta_{0i}-\boldsymbol{\beta} \boldsymbol{\hbox{'}}\;\boldsymbol{x}}}{1+{e}^{\beta_{0i}-\boldsymbol{\beta} \boldsymbol{\hbox{'}}\;\boldsymbol{x}}}, i=2,\kern0.37em 3,\kern0.37em 4 $$

or

3 $$ logit\left(P\left( GWscore\le i\;\Big|\boldsymbol{x}\right)\right)=\kern0.37em  log\frac{P\left( GWscore\le i\;\Big|\boldsymbol{x}\right)}{1-P\left( GWscore\le i\;\Big|\boldsymbol{x}\right)}={\beta}_{0i}-{\boldsymbol{\beta}}^{\boldsymbol{\hbox{'}}}\boldsymbol{x}, i=2,\kern0.37em 3,\kern0.37em 4 $$

where ***x*** is the vector of covariates, *β*_0*i*_ is a parameter that depends on *i*, and ***β*** ' (transposed ***β***) is the coefficient vector which is constant for all *i*. According to equations (2) and (3), there is only one set of coefficients (***β*** ') in the ordinal logistic regression model, and due to the same relationship between each pair of outcome groups, the ordinal logistic model will make the parallel regression assumption [7, 9]. Since only the *β*_0*i*_ differ across values of *i* = 2, 3, 4, the three regression lines are all parallel. The following *Stata* command was used for this model:

$$ ologit\  GWscore\  logCTDI\  id 2\  id 4\ i. patient\ i. observer $$

##### Partial proportional odds model

In situations where the parallel regression assumption is violated, the ordinal logistic regression model is no longer an appropriate model. In this case, an alternative may be the partial proportional odds model, in which some of the *β* coefficients can be the same for all values of *i*, while others can differ (γ_*i*_). Thus, this model is represented in the following form:

4 $$ P\left( GWscore>i\Big|\boldsymbol{x}\right)=\frac{1}{1+{e}^{-{\beta}_{0i}+\boldsymbol{\beta} \boldsymbol{\hbox{'}}\;\boldsymbol{x}+{\gamma}_i^{\mathit{\hbox{'}}}T}},\;i=2,\kern0.37em 3 $$

or

5 $$ logit\ \left(P\left( GWscore>i\;\Big|\boldsymbol{x}\right)\right)={\beta}_{0i}-\boldsymbol{\beta} \mathit{\hbox{'}}\kern0.1em \boldsymbol{x}-{\gamma}_i^{\mathit{\hbox{'}}}T, i=2,\ 3 $$

where ***x*** and *T* are the covariates. This model is more difficult to interpret than the ordinal logistic regression model, since there will be many more parameters to consider and some effects might be statistically insignificant due to the increased number of parameters [6, 10]. We have used the *gologit2* command in *Stata* for this model as follows:

$$ xi\ :\  gologit2\  GWscore\  logCTDI\  id 2\  id 4\  id 4\ i. observer\ i. patient,\  pl\left(i. patient\ \right)\  difficult $$

##### Stereotype logistic model

An alternative model is to consider the response variable as categorical, rather than ordinal, i.e., we are unsure of the relevance of the ordering in the response variable in this case. Also, a multinomial logistic regression model may be suggested when the assumptions of the proportional odds model are not satisfied. Thus, the stereotype ordinal regression model can be considered as imposing ordering constraints on a multinomial model, which is a form of ordinal regression model. Unlike ordered logistic models, stereotype logistic models do not impose the proportional-odds assumption [6, 11]. A full multinomial model can be represented by:

6 $$ P\left( GWscore=s\Big|\boldsymbol{x}\right)=\frac{ \exp \left({\beta}_{0s}-{\boldsymbol{\beta}}_s^{\mathit{\hbox{'}}}\boldsymbol{x}\right)}{{\displaystyle {\sum}_{t=2}^4} \exp \left({\beta}_{0t}-{\boldsymbol{\beta}}_t^{\mathit{\hbox{'}}}\boldsymbol{x}\right)}, $$

where *s* = 2, 3, 4, and *β*_00_ ≡ 0 and *β*_0_ ≡ 0. In the multinomial logistic model, the number of parameter vectors to estimate is *m*-1, where *m* is the number of levels in the response variable. Based on the restriction on the multinomial model by the stereotype logistic model, the number of parameter vectors is between one and min (*m*-1, p), where *p* is the number of covariates [12]. Thus, replacing *β*_*s*_ *= ϕ*_*s*_*β*, the stereotype ordinal regression model can be written as follows:

7 $$ P\left( GWscore=s\Big|\boldsymbol{x}\right)=\frac{ \exp \left({\beta}_{0s}-{\phi}_s\boldsymbol{\beta} \mathit{\hbox{'}}\;\boldsymbol{x}\right)}{{\displaystyle {\sum}_{t=2}^4} \exp \left({\beta}_{0t}-{\phi}_t\boldsymbol{\beta} \mathit{\hbox{'}}\;\boldsymbol{x}\right)}, $$

where *β*_00_ = *ϕ*_0_≡0. This was achieved with the following *Stata* command:

$$ slogit\  GWscore\  logCTDI\  id 2\  id 4\ i. patient\ i. observer $$

#### Regression models with random effects

In this section, it is supposed that three covariates including log(*CTDI*), *id*2 and *id*4 are considered as fixed effects and two covariates including *patient* and *observer* are specified as crossed random effects. The basic concept of a random effects model is that the variation across entities is assumed to be random and uncorrelated with the covariates, unlike the fixed effects model. The mixed linear model as well as the mixed-effects ordered logistic regression model will be discussed to analyze the data when there are both fixed and random effects in the model.

##### Mixed linear model

The simplest model to analyze a data set with both fixed effects and random effects is a mixed linear model, which can be written in the following form:

8 $$ GWscore=\boldsymbol{\beta} \boldsymbol{\hbox{'}}\boldsymbol{x}+\boldsymbol{b}\boldsymbol{\hbox{'}}\boldsymbol{z}+\epsilon, $$

where ***x*** is the model matrix for *id*2, *id*4 and *nd* as fixed effects, ***z*** is the model matrix for *patient* and *observer* as random effects, ***β*** is the vector of fixed-effects coefficients, ***b*** is the vector of random-effects coefficients, and *ϵ* is an error term [13]. We have used the *mixed* command in *Stata* for a mixed linear model including crossed random effects as follows:

$$ mixed\  GWscore\  id 2\  id 4\  logCTDI\ \left|\right|\ \_ all:\ R. observer\left|\right|\_ all:\ R. patient $$

##### Mixed-effects ordered logistic regression

A model that can handle random effects where the response variable is ordinal is the mixed-effects ordered logistic regression [14]. In contrast to the ordinal logistic model, the model with random effects has the form:

9 $$ P\left( GWscor{e}_{ij}\le t\;\Big|\;{\boldsymbol{x}}_{ij},{\boldsymbol{z}}_{ij}\right)=\frac{ \exp \left({\alpha}_t-\boldsymbol{\beta} \mathit{\hbox{'}}{\boldsymbol{x}}_{ij}-{\boldsymbol{u}}_i^{\mathit{\hbox{'}}}{\boldsymbol{z}}_{ij}\right)}{1+ \exp \left({\alpha}_t-\boldsymbol{\beta} \mathit{\hbox{'}}{\boldsymbol{x}}_{ij}-{\boldsymbol{u}}_i^{\mathit{\hbox{'}}}{\boldsymbol{z}}_{ij}\right)},\ t=2,\;3, $$

or

10 $$ logit\ P\left( GWscor{e}_{ij}\le t\;\Big|\;{\boldsymbol{x}}_{ij},{\boldsymbol{z}}_{ij}\right)={\alpha}_t-\boldsymbol{\beta} \mathit{\hbox{'}}{\boldsymbol{x}}_{ij}-{\boldsymbol{u}}_i^{\mathit{\hbox{'}}}{\boldsymbol{z}}_{ij}, $$

where **z**_*ij*_ refers to a vector of covariates for the random effects (*patient* and *observer*) and ***u***_*i*_ is the vector of random-effects coefficients [14]. In *Stata*, the *meologit* command can be used for the ordinal logistic regression model with crossed random effects as follows:

$$ meologit\  GWscore\  logCTDI\  id 2\  id 4\ \left|\right|\ \_ all:\ R. observer\left|\right|\ \_ all:\ R. patient $$

##### Goodness of fit

The metrics used to compare the methods were the pseudo *R*^2^ and Akaike’s information criterion (AIC). The *Pseudo R*^2^, also called McFadden’s *R*^2^, [15], defined by

11 $$ {R}_{McF}^2=1-\frac{ \log\ \widehat{L}\left({M}_{Full}\right)}{ \log\ \widehat{L}\left({M}_{intercept}\right)} $$

is one of several approximations of the *R*^2^ for linear regression. None of these are interpreted as the *R*^2^ for linear regression, and they all give different result [16]. An advantage of the McFadden *R*^2^, in addition to its simple definition, is that it can be used for all models estimated by maximum likelihood. Since all models used in this study are based on maximum likelihood, the McFadden *R*^2^ is calculated in the same way for all models, and they can therefore be compared with respect to *R*^2^. The model with the largest *R*^2^ is the one that best fits the data.

However, for comparing models differing in the number of parameters, AIC [17] is more suitable:

12 $$ AIC=\frac{-2\  \log\ \widehat{L}\left({M}_k\right)+2p}{N} $$

The most common alternative to AIC is the Bayesian information criterion (BIC). However, BIC takes the number of parameters (the degrees of freedom) into account in a way that makes it less appropriate than AIC for selecting between models with different number of parameters. The model with the smallest AIC value is considered to be the best [17].

##### Estimation of potential for dose reduction

To estimate the dose reduction (in percent) that might come about by the application of *id*2 and *id*4, we have used the technique proposed in our earlier publication [3], which relates the effect of replacing the reconstruction method to that of changing the effective dose. This involves forming the ratio between two regression coefficients and computing the confidence limits of the final expression using the delta method [18]. The required *Stata* commands to be applied after fitting the regression model are as follows:

$$ nlcom\ \left( dosereduction\_ id 2:\  1- exp\left(-\left(\_b\left[ id 2\right]/\_b\left[ logCTDI\right]\right)\right)\right) $$

$$ nlcom\ \left( dosereduction\_ id 4:\  1- exp\left(-\left(\_b\left[ id 4\right]/\_b\left[ logCTDI\right]\right)\right)\right) $$

### Analysis of ranking data

Rank-order data differ in certain respects from grading data where each case is graded on the same absolute scale. One way of understanding ranking is to regard it as a sequence of choices. Then, there is gradually less freedom in the choice of grades, since the earlier choices constrain the available ranks for subsequent cases to those not used previously. This motivates the introduction of dedicated regression techniques for situations with rank-order data.

All regression models discussed in the previous section (including the linear model, ordinal logistic regression, partial proportional odds model, stereotype logistic model, mixed linear model and mixed-effects ordered logistic regression) can be applied to the data in which the response variable is *GWrank*. Besides these regression models, the rank-ordered logistic regression model can be an appropriate model since there is some extra information about the ranking of outcomes.

We define the response of respondent *i* by the vector *y*_*i*_ = (*y*_*i*1_, …, *y*_*iJ*_)^'^, where *y*_*ij*_ denotes the rank that individual *i* gives to item *j*. Let *GWrank*_*ij*_ = 1 represent the event that respondent *i* most prefers alternative *j*. This leads to the following expression for the probability that item *j* is most preferred by individual *i*:

13 $$ P\left( GWran{k}_{ij}=1\;\Big|\;{\boldsymbol{x}}_{ij}\right)=\frac{ \exp\;\left({\boldsymbol{x}}_i^{\mathit{\hbox{'}}}{\beta}_j\right)}{{\displaystyle {\sum}_{t=1}^J} \exp\;\left({\boldsymbol{x}}_i^{\mathit{\hbox{'}}}{\beta}_t\right)} $$

where *β* = {*β*_1_, …, *β*_*J*_} and *β*_*J*_ is considered as zero for identification [19]. We have used the *rologit* command in *Stata*, which is specifically designed for ranking data, as follows:

$$ rologit\  GWrank\  logCTDI\  id 2\  id 4,\ group(groupid) $$

where *groupid* is an identifier variable that links the alternatives. Since the default for the *rologit* command is that higher values represent more attractive alternatives, we have recoded the *GWrank* variable to have a higher value indicating better quality. In this case, the *Stata* output is the same as when the *reverse* option in *rologit* is used, which specifies that in the preference order, a higher number means a less attractive alternative in the original data [10].

## Results

### Absolute scores

The results of the different regression models for *GWscore*, *BGscore* and *GQscore* are presented in Tables 1, 2 and 3, respectively. The intercepts are excluded from the reported results in these tables due to different parameterization of the regression models. The analyses have been made using fixed effects models as well as fixed and random effects models, as explained in the previous section. All regression coefficients are statistically significant at the 0.01 level, except when contrasting categories 1 & 2 with category 3 (highest image quality) in the second panel with the partial proportional odds model for *id*2 with *GWscore* (Table 1), and for both *id2* and *id4* with *BGscore* and *GQscore* (Tables 2 and 3). The confidence intervals of the coefficients are reported in parentheses in Tables 1, 2 and 3.

**Table 1:** Estimated parameters, goodness-of-fit statistics and estimated dose reduction for GWscore

Model	Coefficient						Goodness-of-fit		Dose reduction	
	logCTDI		id2		id4		AIC	Pseudo R^2^	id2	id4
	Est.	P-value	Est.	P-value	Est.	P-value				
regress^a^	1.459	<0.001	0.158	<0.001	0.208	<0.001	-	0.4160	10.29 %	13.31 %
	(1.244, 1.674)		(0.082, 0.234)		(0.132, 0.284)				(6.14 %, 14.43 %)	(9.37 %, 17.24 %)
ologit^a^	8.825	<0.001	0.966	<0.001	1.271	<0.001	1124.35	0.4172	10.37 %	13.41 %
	(7.354, 10.295)		(0.512, 1.419)		(0.812, 1.730)				(6.35 %, 14.39 %)	(9.60 %, 17.23 %)
gologit2^a^ =2	9.487	<0.001	1.262	<0.001	1.465	<0.001	1184.56	0.4342	12.45 %	14.31 %
	(7.213, 11.761)		(0.682, 1.842)		(0.873, 2.058)				(7.44 %, 17.46 %)	(9.25 %, 19.37 %)
gologit2^a^ =3	8.165	<0.001	0.521	0.172	0.985	0.008			6.18 %	11.37 %
	(6.143, 10.189)		(−.227, 1.269)		(0.260, 1.711)				(−1.62 %, 13.98 %)	(4.87 %, 17.86 %)
slogit^a^	17.447	<0.001	1.887	<0.001	2.433	<0.001	1123.27	0.4201	10.25 %	13.05 %
	(14.460, 20.435)		(1.028, 2.746)		(1.555, 3.310)				(6.35 %, 14.15 %)	(9.23 %, 16.80 %)
mixed^b^	1.459	<0.001	0.158	<0.001	0.208	<0.001	1225.30	0.2748	10.29 %	13.31 %
	(1.244, 1.673)		(0.082, 0.234)		(0.132, 0.284)				(6.14 %, 14.43 %)	(9.38 %, 17.24 %)
meologit^b^	8.433	<0.001	0.922	<0.001	1.213	<0.001	1215.96	0.2751	10.36 %	13.4 %
	(6.685, 10.180)		(0.452, 1.392)		(0.735, 1.692)				(6.21 %, 14.51 %)	(9.49 %, 17.32 %)

95 % confidence limits of each estimate given in parentheses

^a^regression model with fixed effects only

^b^regression model with fixed and random effects

**Table 2:** Estimated parameters, goodness-of-fit statistics and estimated dose reduction for BGscore

Model	Coefficient						Goodness-of-fit		Dose reduction	
	logCTDI		id2		id4		AIC	Pseudo R^2^	id2	id4
	Est.	P-value	Est.	P-value	Est.	P-value				
regress^a^	1.329	<0.001	0.129	0.001	0.183	<0.001	-	0.3645	9.26 %	12.88 %
	(1.113, 1.546)		(0.052, 0.206)		(0.107, 0.260)				(4.58 %, 13.94 %)	(8.50 %, 17.26 %)
ologit^a^	8.249	<0.001	0.760	0.001	1.071	<0.001	1135.11	0.3705	8.80 %	12.17 %
	(6.766, 9.732)		(0.321, 1.200)		(0.623, 1.520)				(4.44 %, 13.17 %)	(8.00 %, 16.35 %)
gologit2^a^	7.804	<0.001	0.883	0.001	1.431	<0.001	1190.70	0.3915	10.69 %	16.76 %
=2	(5.807, 9.801)		(0.368, 1.398)		(0.877, 1.986)				(5.18 %, 16.21 %)	(10.97 %, 22.55 %)
gologit2^a^	7.842	<0.001	0.505	0.261	0.408	0.369			6.24 %	5.07 %
=3	(5.577, 10.107)		(−.376, 1.387)		(−.481, 1.298)				(−3.27 %, 15.76 %)	(−4.86 %, 15.00 %)
slogit^a^	15.378	<0.001	1.337	0.001	2.036	<0.001	1124.99	0.3791	8.33 %	12.40 %
	(12.340, 18.42)		(0.577, 2.098)		(1.246, 2.826)				(4.17 %, 12.48 %)	(8.30 %, 16.50 %)
mixed^b^	1.329	<0.001	0.129	0.001	0.183	<0.001	1224.58	0.2207	9.26 %	12.88 %
	(1.114,1.545)		(0.053, 0.206)		(0.107, 0.260)				(4.59 %, 13.93 %)	(8.51 %, 17.26 %)
meologit^b^	7.806	<0.001	0.736	<0.001	1.031	<0.001	1216.93	0.2230	9.00 %	12.38 %
	(6.733, 8.879)		(0.327, 1.146)		(.618, 1.445)				(4.52 %, 13.48 %)	(8.09 %, 16.67 %)

95 % confidence limits of each estimate given in parentheses

^a^regression model with fixed effects only

^b^regression model with fixed and random effects

**Table 3:** Estimated parameters, goodness-of-fit statistics and estimated dose reduction for GQscore

Model	Coefficient						Goodness-of-fit		Dose Reduction	
	logCTDI		id2		id4		AIC	Pseudo R^2^	id2	id4
	Est.	P-value	Est.	P-value	Est.	P-value				
regress^a^	1.424	<0.001	0.158	<0.001	0.175	<0.001	-	0.3560	10.53 %	11.57 %
	(1.217, 1.630)		(0.085, 0.232)		(0.102, 0.248)				(6.46 %, 14.59 %)	(7.58 %, 15.56 %)
ologit^a^	9.626	<0.001	1.011	<0.001	1.133	<0.001	1060.25	0.3573	9.97 %	11.10 %
	(8.020, 11.232)		(0.547, 1.476)		(0.665, 1.600)				(6.13 %, 13.17 %)	(7.32 %, 14.88 %)
gologit2^a^	8.627	<0.001	1.092	<0.001	1.484	<0.001	1113.99	0.3816	11.89 %	15.80 %
=2	(6.519, 10.735)		(0.558, 1.625)		(0.924, 2.044)				(6.74 %, 17.03 %)	(10.48 %, 21.12 %)
gologit2^a^ =3	9.652	<0.001	0.964	0.073	0.387	0.507			9.50 %	3.93 %
	(6.915, 12.388)		(−0.091, 2.019)		(−0.756, 1.529)				(1.29 %, 17.72 %)	(−6.76 %, 14.61 %)
slogit^a^	18.523	<0.001	1.867	<0.001	2.148	<0.001	1061.13	0.3594	9.59 %	10.95 %
	(15.277,21.769)		(0.997, 2.738)		(1.275, 3.022)				(5.80 %, 13.38 %)	(7.23 %, 14.67 %)
mixed^b^	1.424	<0.001	0.158	<0.001	0.175	<0.001	1119.97	0.1857	10.53 %	11.57 %
	(1.218, 1.630)		(0.085, 0.231)		(0.102, 0.248)				(6.47 %, 14.59 %)	(7.58 %, 15.55 %)
meologit^b^	9.179	<0.001	0.967	<0.001	1.085	<0.001	1123.66	0.1853	10.00 %	11.15 %
	(7.031, 11.328)		(0.468, 1.467)		(0.578, 1.592)				(6.03 %, 13.98 %)	(7.25 %, 15.05 %)

95 % confidence limits of each estimate given in parentheses

^a^regression model with fixed effects only

^b^regression model with fixed and random effects

In the linear model (*regress*), the regression equation of *GWscore* can be obtained using the coefficients reported in Table 1. The relationship between the covariates and the response variable is assumed to be linear, and an increase in the independent variables – i.e. increasing the effective dose as well as replacing the standard reconstruction with *id2* or *id4* – results in an increase in the *GWscore*, since the signs of all regression coefficients are positive.

In the ordinal logistic model (*ologit*), a log(*CTDI*) coefficient of 8.825 implies that a doubling of the *CTDI* for one of the image stacks in the comparison would lead to a huge increase by a factor of 2^8.825^ = 453.513 in the odds for a higher score for that stack. The coefficient regression for *id*2 and *id*4 are 0.966 and 1.271, respectively, and they can be interpreted to the odds being multiplied by *e*^0.966^ = 2.627 and *e*^1.271^ = 3.564, respectively, when the corresponding iterative reconstruction method is used instead of the standard method.

For the partial proportional logistic model (*gologit2*), the first panel contrasts *GWscore* = 1 with categories 2 and 3, whereas the second panel contrasts with category 4. Hence, positive coefficients indicate that higher values on the independent variable make it more likely that the respondent will be in a higher category of *GWscore* than the current one.

Since the stereotype model (*slogit*) is a type of an ordinal logistic regression model, the interpretation of its coefficients is similar to the ordinal logistic model. For the *id*2 and *id*4 variables, the odds of the highest image quality versus lowest image quality increased by a factor of e^1.887^ = 6.6 and e^2.433^ = 11.4, respectively, holding all other variables constant. As discussed in the previous section, there is another parameter in the stereotype model and that is *ϕ*_s_. Since the response variable has only three categories in this case, it is supposed that *ϕ*_0_≡0, *ϕ*_2_≡1, and the estimate of *ϕ*_1_ is equal to 0.431. Since we have *ϕ*_0_ < *ϕ*_1_ < *ϕ*_2_, we conclude that the stereotype logistic model confirms that the subjective assessment of the dependent variable is indeed ordered, and the groups (*GWscore* categories) are distinguishable.

For the mixed linear model (*mixed*), the regression coefficients are similar to the linear regression model with fixed effects (*regress*) and the only difference is that the *patient* and *observer* variables have been considered as random effects in the mixed linear model.

Also the regression coefficients of the mixed-effects ordered logistic regression (*meologit*) are very close to the ordinal logistic regression model (*ologit*). The estimates of the variance of the random intercept at the *observer* and *patient* level are 0.689 and 4.478, respectively.

The goodness-of-fit statistics (*AIC* and *Pseudo R*^2^) of all regression models are also given in Tables 1, 2 and 3. In Tables 1 and 2 the *slogit* model, and in Table 3 the *ologit* model, present the smallest *AIC* among all fixed effects models, although the differences are small. The *gologit2* model represents the largest *Pseudo R*^*2*^ among all fixed effects models in Tables 1, 2 and 3.

The estimated potential for reduction of the *CTDI* settings (dose reduction) for *GQscore*, *BGscore* and *GQscore* are reported in Tables 1, 2 and 3, respectively. The confidence limits of the dose reductions, calculated using the delta method, are also presented. The proposed percentage of dose reduction for *id*2 (around 10 %, with confidence intervals around (6 %, 14 %), for *GWscore*) is very similar for all regression models in Table 1, except for the partial proportional odds model. This is also true for the estimated percentages of dose reduction for *id*4 (around 13 %, with a confidence interval around 9 %, 17 %). The results thus indicate smaller dose reductions for *id*2 than for *id*4, although the confidence intervals overlap to a large extent.

To compare the effect of *id*2 with *id*4 on the response variable, we restricted the analysis to observations using *id2* or *id4* and considered only one covariate (*id*2) in the regression models. The estimates thus obtained and their confidence intervals are reported in Table 4 for *GWscore*, *BGscore* and *GQscore*. It was found that the coefficients are all statistically insignificant at the 0.01 level.

**Table 4:** Parameter estimation of id2 versus id4

Model	GWscore		GQscore		BGscore	
	Est.	*P*-value	Est.	*P*-value	Est.	*P*-value
regress^a^	−0.050	0.199	−0.017	0.641	−0.054	0.141
	(−0.126, 0.026)		(−0.087, 0.054)		(−0.126, 0.018)	
ologit^a^	−0.322	0.164	−0.13	0.603	−0.374	0.127
	(−0.775, 0.131)		(−0.621, 0.361)		(−0.854, 0.107)	
gologit2^a^	−0.215	0.488	−0.393	0.185	−0.596	0.046
=2	(−0.823, 0.392)		(−0.975, 0.189)		(−1.182, −0.010)	
gologit2^a^	−0.472	0.182	0.629	0.228	0.104	0.82
=3	(−1.166, 0.221)		(−0.394, 1.653)		(−0.788, 0.996)	
slogit^a^	−0.592	0.194	−0.408	0.281	−0.743	0.052
	(−1.485, 0.301)		(−1.150, 0.334)		(−1.491, 0.005)	
mixed^b^	−0.050	0.176	−0.017	0.640	−0.054	0.14
	(−0.122, .022)		(−0.087, 0.053)		(−0.126, 0.018)	
meologit^b^	−0.3217	0.164	−0.126	0.598	−0.336	0.152
	(−0.775, 0.131)		(−0.597, 0.344)		(−0.794, 0.123)	

95 % confidence limits given in parentheses

^a^regression model with fixed effects only

^b^regression model with fixed and random effects

### Ranking data

The rank-ordered logistic regression model was applied with *GWrank*, which represents the ranked order between the four imaging protocols, as the response variable. The regression coefficients, goodness-of-fit statistics and the estimates of dose reduction for linear models (fixed effects and mixed effects), ordinal logistic regression models (fixed effects and mixed effects) as well as the rank-ordered logistic model are reported in Table 5. All regression coefficients are statistically significant at the 0.01 level. The rank-ordered logistic model, which is designed specifically for analyzing rank-order data, presents the best performance among all models in terms of the goodness-of-fit measures (*AIC* and *Pseudo R*^*2*^). Unlike the results of *GWscore*, the estimated dose reduction figures for *id*2 (around 18 %) were greater than for *id*4 (around 15 %) while working with *GWrank*. The corresponding finding was also made for *BGrank* and *GQrank*. (Tables 6 and 7) In all cases, though, there was considerable overlap of the confidence intervals.

**Table 5:** Estimated parameters, Goodness-of-fit statistics and dose reduction of GWrank

Model	Coefficient						Goodness-of-fit		Dose Reduction	
	logCTDI		id2		id4		AIC	Pseudo R^2^	id2	id4
	Est.	P-value	Est.	P-value	Est.	P-value				
regress^a^	4.482	<0.001	0.850	<0.001	0.696	<0.001	-	0.0959	17.27 %	14.38 %
	(3.981, 4.983)		(0.673, 1.027)		(0.518, 0.873)				(14.44 %, 20.11 %)	(11.44 %, 17.32 %)
ologit^a^	9.247	<0.001	1.780	<0.001	1.531	<0.001	2462.68	0.1138	17.51 %	15.26 %
	(8.126, 10.368)		(1.428, 2.132)		(1.168, 1.894)				(14.99 %, 20.02 %)	(12.51 %, 18.01 %)
rologit^a^	5.537	<0.001	1.232	<0.001	0.932	<0.001	1303.66	0.1493	19.95 %	15.49 %
	(4.734, 6.340)		(0.969, 1.495)		(0.666, 1.197)				(17.06 %, 22.84 %)	(12.24 %, 18.74 %)
mixed^b^	4.482	<0.001	0.850	<0.001	0.696	<0.001	2670.66	0.0000	17.27 %	14.38 %
	(3.995, 4.970)		(0.677, 1.023)		(0.523, 0.869)				(14.51 %, 20.04 %)	(11.51 %, 17.24 %)
meologit^b^	9.267	<0.001	1.751	<0.001	1.549	<0.001	2452.89	0.0320	17.22 %	15.40 %
	(8.991, 9.543)		(1.416, 2.086)		(1.214, 1.885)				(14.36 %, 20.07 %)	(12.43 %, 18.36 %)

95 % confidence limits of each estimate given in parentheses

^a^regression model with fixed effects only

^b^regression model with fixed and random effects

**Table 6:** Estimated parameters, Goodness-of-fit statistics and dose reduction of BGrank

Model	Coefficient						Goodness-of-fit		Dose Reduction	
	logCTDI		id2		id4		AIC	Pseudo R^2^	id2	id4
	Est.	P-value	Est.	P-value	Est.	P-value				
regress^a^	−4.812	<0.001	−0.863	<0.001	−0.683	<0.001	-	0.1141	16.41 %	13.24 %
	(−5.299, −4.324)		(−1.035, −0.690)		(−0.856, −0.511)				(13.82 %, 19.00 %)	(10.53 %, 15.95 %)
ologit^a^	−9.793	<0.001	−1.734	<0.001	−1.424	<0.001	2420.57	0.1297	16.23 %	13.53 %
	(−10.916, −8.671)		(−2.081, −1.387)		(−1.780, −1.067)				(13.80 %, 18.65 %)	(10.86 %, 16.20 %)
rologit^a^	−5.344	<0.001	−0.881	<0.001	−0.836	<0.001	1308.64	0.1461	15.20 %	14.48 %
	(−6.116, −4.571)		(−1.117. −0.645)		(−1.081, −0.592)				(12.01 %, 18.40 %)	(11.10 %, 17.87 %)
mixed^b^	−4.812	<0.001	−0.862	<0.001	−0.683	<0.001	2617. 17	0.000	16.41 %	13.24 %
	(−5.286, −4.337)		(−1.030, −0.694)		(−0.851, −0.515)				(13.88 %, 18.94 %)	(10.60 %, 15.88 %)
meologit^b^	−10.460	<0.001	−1.861	<0.001	−1.545	<0.001	2355.74	0.0086	16.30 %	13.73 %
	(−10.633, −10.288)		(−2.144, −1.579)		(−1.846, −1.245)				(14.01 %, 18.59 %)	(11.24 %, 16.23 %)

95 % confidence limits of each estimate given in parentheses

^a^regression model with fixed effects only

^b^regression model with fixed and random effects

**Table 7:** Estimated parameters, Goodness-of-fit statistics and dose reduction of GQrank

Model	Coefficient						Goodness-of-fit		Dose Reduction	
	logCTDI		id2		id4		AIC	Pseudo R^2^	id2	id4
	Est.	P-value	Est.	P-value	Est.	P-value				
regress^a^	−4.671	<0.001	−0.817	<0.001	−0.463	<0.001	-	0.1134	16.04 %	9.43 %
	(−5.158, −4.183)		(−.989, −.644)		(−0.635, −0.290)				(13.36 %, 18.73 %)	(6.44 %, 12.42 %)
ologit^a^	−9.433	<0.001	−1.691	<0.001	−1.033	<0.001	2427.97	0.1269	16.41 %	10.37 %
	(−10.550, −8.317)		(−2.039, −1.343)		(−1.395, −0.672)				(13.93 %, 18.88 %)	(7.39 %, 13.36 %)
rologit^a^	−4.766	<0.001	−0.709	<0.001	−0.576	<0.001	1337.79	0.1270	13.81 %	11.38 %
	(−5.516, −4.015)		(−0.940, −0.477)		(−0.823, −0.328)				(10.23 %, 17.40 %)	(7.35 %, 15.41 %)
mixed^b^	−4.671	<0.001	−0.8167	<0.001	−0.4625	<0.001	2619.45	0.0000	16.04 %	9.43 %
	(−5.145, −4.196)		(−0.985, −0.648)		(−0.631, −0.294)				(13.42 %, 18.66 %)	(6.51 %, 12.34 %)
meologit^b^	−9.380	<0.001	−1.680	<0.001	−1.027	<0.001	2367.96	0.0105	16.40 %	13.37 %
	(−9.506, −9.255)		(−1.956, −1.405)		(−1.331, −0.723)				(13.91 %, 18.89 %)	(7.45 %, 13.29 %)

95 % confidence limits of each estimate given in parentheses

^a^regression model with fixed effects only

^b^regression model with fixed and random effects

## Discussion

In the present study, we did not find any dramatic differences in the results between the tested regression models. Overall, the goodness-of-fit statistics in Tables 1, 2 and 3 were similar in magnitude for all the tested models, with the exception of the *Pseudo R*^*2*^ values for the mixed effects models (*mixed* and *meologit*), which were considerably lower than for the models with fixed effects only. This is most likely due to the different numbers of parameters in the models. However, also with *AIC*, which is supposed to compensate for differences in the number of fitted parameters, slightly worse fit was found for the models including random effects.

The original study using the same data [8] applied a linear mixed model, corresponding to the analysis here described by the command *mixed*. The findings were basically the same in the new analysis, with significant differences between the normal dose reconstructions and all other schemes, as well as significant effects of the iterative algorithms applied to reduced-dose data, for all the tested image quality criteria. In this study, we also added the estimation of potential dose reductions, which is important for clinical application of the results.

As for the regression coefficients, their values from the linear models should not be directly compared with those from the logistic models, due to entirely different principles for parametrization. It may be noted, though, that the addition of random effects in the linear models (*mixed* vs. *regress*) had no effect on the coefficient estimates and hardly any on the confidence limits. Among the logistic models, the most striking finding was the fact that with *gologit2*, different estimates were obtained when contrasting the two best categories than when contrasting the two worst categories (second vs. first *gologit2* panel in Tables 1, 2 and 3). This suggests that the proportional odds assumption may not have been appropriate for these data. To test this, the commonly recommended procedure is to apply Brant’s test [20]. Unfortunately, the *Stata* implementation of Brant’s test (which only works with *ologit*) does not allow nominal or random effects, so we were not able to carry out a formal test of this assumption. Also, when comparing the logistic models *ologit* and *meologit*, the addition of random effects had only a minute effect on the estimates. It should be kept in mind that when there are two crossed random effects in the model (in this case *patient* and *observer*), the integration method used by *Stata* is Laplacian integration, in which the parameter estimates are biased. In the variance components, the bias of the estimates tends to be more prominent than in the estimates of the fixed effects due to the Laplacian approximation [14].

For all the tested models (except *gologit2* at the highest level), the regression coefficients had larger values for *id4* than for *id2*, which was expected from previous knowledge about the algorithms, with *id4* differing more from the standard algorithm than *id2*. The confidence intervals, though, overlapped to a large extent. The difference between *id4* and *id2* was also not significant when tested formally (Table 4).

More interesting from an application point of view are probably the estimates of potential dose reductions. Here all the regression models that summarize the different image quality levels gave similar results for the three image quality scores, with somewhat larger estimates for *id4* than for *id2*, as expected, but widely overlapping confidence intervals. For *gologit2*, contrasting the highest quality levels gave smaller estimates than contrasting the lowest levels for both *id4* and *id2*. A possible interpretation is that it will be more difficult to maintain the probability of producing images of excellent quality by applying the new reconstruction algorithms while reducing the radiation dose than to maintain the probability of producing images of clinically acceptable quality. Thus, the somewhat different results for the two levels seem, to some extent, to answer different research questions. The fact that, in general, non-significant results were obtained when contrasting the highest quality levels may be related both to the weaker effect at this level and to a loss of power when more parameters are estimated from the same data.

When analyzing the rank-order data (Tables 5–7), the regression model specifically designed for this type of data, *rologit*, yielded much better fit (lower *AIC* and higher *Pseudo R*^*2*^). A surprising finding was that with the ranking data, larger effects, and thus larger dose reduction estimates, were found for *id2* than for *id4*. The difference was even greater with *rologit*. However, again the two confidence intervals overlap.

Broadly speaking, the results of our comparison did not give any clear-cut empirical evidence for selecting the most appropriate regression model for analyzing visual grading data in medical imaging, except for choosing *rologit* when analyzing rank data. Thus, the selection of model must be based on other considerations.

The use of linear models for analyzing ordinal scale data is generally discouraged in statistical textbooks. Also, on theoretical grounds, it is commonly recommended to handle variables such as *patient* and *observer* in our study as random effects, since they both represent samples from larger populations. This would speak in favor of the *meologit* approach when analyzing absolute scores. The greatest problem of this model appears to be the proportional odds assumption (parallel regression assumption), which may well have been violated by our data. Using instead *gologit2* might resolve this problem, but at the expense of more complex results that are less straightforward to interpret. Still, there are situations where the relevant research questions may motivate this more complex model. It is more difficult to weigh the importance of handling violations of the proportional odds assumption (*gologit2*) against correctly controlling random effects (*meologit*). Also for *slogit*, the results are more complex and possibly difficult for an applied researcher to interpret. The main finding from *slogit* in our study was the confirmation of the ordinal structure that had been defined beforehand.

## Conclusions

In conclusion, a number of logistic regression methods are available for handling ordinal data from visual grading experiments in medical imaging. Our study did not provide any empirical support for selecting a different regression model than the one we would recommend on theoretical grounds, i.e. the ordinal logistic regression model with mixed effects, which is appropriate for handling random effects when the response variable is ordinal. For rank-order data, the rank-ordered logistic regression model appears to be most appropriate, since this model can handle the rank-order data correctly and because of its better performance in terms of the goodness-of-fit among the tested regression models.

## Abbreviations

AIC: Akaike information criterion
ANOVA: Analysis of variance
BG: Basal ganglia delineation
CT: Computed tomography
CTDI_vol_: Volume computed tomography dose index
fd: Full dose
gologit2: Generalized ordered logit/partial proportional odds
GQ: General image quality
GW: Gray-white-matter discrimination
id2: Iterative reconstruction with noise reduction level 2
id4: Iterative reconstruction with noise reduction level 4
meologit: Mixed-effects ordered logistic regression
ologit: Ordinal logistic regression
rd: Reduced dose
ROC: Receiver operating characteristic
rologit: Rank-ordered logistic regression
slogit: Stereotype logistic regression
